# Molecular Study of the Protective Effect of a Low-Carbohydrate, High-Fat Diet against Brain Insulin Resistance in an Animal Model of Metabolic Syndrome

**DOI:** 10.3390/brainsci13101383

**Published:** 2023-09-28

**Authors:** Abdulhadi Bima, Basmah Eldakhakhny, Aliaa A. Alamoudi, Zuhier Awan, Abrar Alnami, Salwa Mohamed Abo-Elkhair, Hussein Sakr, Fatma Mohamed Ghoneim, Ayman Elsamanoudy

**Affiliations:** 1 Clinical Biochemistry Department, Faculty of Medicine, King Abdulaziz University, Jeddah 21465, Saudi Arabia; hadibima@hotmail.com (A.B.); beldakhakhny@kau.edu.sa (B.E.); aaalamoudi2@kau.edu.sa (A.A.A.); zawan@kau.edu.sa (Z.A.); abraralnami@outlook.com (A.A.); 2 Food, Nutrition, and Lifestyle Research Unit, King Fahd for Medical Research Centre, King Abdulaziz University, Jeddah 21465, Saudi Arabia; 3 Medical Biochemistry and Molecular Biology Department, Faculty of Medicine, Mansoura University, Mansoura 35516, Egypt; salwakhair@mans.edu.eg; 4 Physiology Department, College of Medicine and Health Sciences, Sultan Qaboos University, Muscat 123, Oman; hsakr@squ.edu.om; 5 Medical Physiology Department, Faculty of Medicine, Mansoura University, Mansoura 35516, Egypt; 6 Faculty Development Unit, Physiological Science and Medical Education Department, Fakeeh College for Medical Sciences, Jeddah 23323, Saudi Arabia; fghoneim@fcms.edu.sa

**Keywords:** metabolic syndrome, brain insulin resistance, S100B, BDNF, TNF-α, IGF-1, IGF-1R, IGFBP-2, IGFBP-5, Bax, Bcl-2, caspase-3, low carbohydrate-high fat diet

## Abstract

Brain insulin resistance is linked to metabolic syndrome (MetS). A low-carbohydrate, high-fat (LCHF) diet has been proposed to have a protective effect. Therefore, this study aimed to investigate the brain insulin resistance markers in a rat animal model of MetS and the protective effects of the LCHF diet. Four groups of male rats (10/group) were created. Group I (Control) was fed a regular diet. Groups II–IV were injected with dexamethasone (DEX) to induce MetS. Group II received DEX with a regular diet. Group III (DEX + LCHF) rates were fed a low-carbohydrate, high-fat diet, while Group IV (DEX + HCLF) rats were fed a high-carbohydrate, low-fat (HCLF) diet. At the end of the four-week experiment, HOMA-IR was calculated. Moreover, cerebral gene expression analysis of S-100B, BDNF, TNF-α, IGF-1, IGF-1 R, IGFBP-2, IGFBP-5, Bax, Bcl-2, and caspase-3 was carried out. In the DEX group, rats showed a significant increase in the HOMA-IR and a decrease in the gene expression of IGF-1, IGF-1 R, IGFBP-2, IGFBP-5, BDNF, and Bcl2, with a concomitant rise in S100B, TNF-α, Bax, and caspase-3. The LCHF diet group showed a significantly opposite effect on all parameters. In conclusion, MetS is associated with dysregulated cerebral gene expression of BDNF, S100B, and TNF-α and disturbed IGF-1 signaling, with increased apoptosis and neuroinflammation. Moreover, the LCHF diet showed a protective effect, as evidenced by preservation of the investigated biochemical and molecular parameters.

## 1. Introduction

The brain is a complex organ that utilizes 20% of the body’s glucose despite its small mass, just 2% of body weight [1]. Although glucose utilization is not dependent on insulin-stimulated translocation of glucose transporter 4 (GLUT4), the brain was labeled as an insulin-sensitive organ early. The brain’s insulin sensitivity comes from the widespread expression of the insulin receptor in the brain. In addition, it is now apparent that insulin and its close relative, insulin-like growth factor 1 (IGF1), can influence brain metabolism and cellular function [2]. IGF1 plays a pivotal role in brain development and maintenance. It promotes neuronal survival, differentiation, and myelination. In adults, IGF1 is linked to cognitive function and neurogenesis. It acts through the IGF1 receptor, influencing brain glucose uptake and utilization differently from insulin [3].

Insulin receptor pathways, commonly observed in peripheral systems, play a significant role in various functions within the central nervous system [4].

Insulin receptors are located ubiquitously throughout the brain, and their expression is high in specific regions, such as the cerebellum, cortex, and hypothalamus [5]. In general, insulin’s normal physiological action via its own receptors plays a crucial role in the development of the nervous system, influencing the differentiation, proliferation, and growth of nerve cells. Insulin exerts its effects by binding to its receptor, leading to increased expression and activity of insulin in the developing brain tissues, including neurons and glial cells. Additionally, insulin is a neuroprotective agent that protects against damage caused by ischemia, β-amyloid toxicity, oxidative stress, and apoptosis [6].

Reduced sensitivity of body tissues to insulin action is known as insulin resistance (IR). The lack of receptor response to insulin could be due to the downregulation of insulin receptors, the inability of insulin receptors to bind to insulin, or faulty activation of the insulin signaling cascade. Likewise, the failure of brain cells to respond to insulin is known as brain IR [7]. Brain IR can lead to metabolic disruptions, such as overeating and impaired glucose utilization. It is also associated with cognitive challenges, including memory loss, reduced flexibility, and increased risk for diseases such as Alzheimer’s. Furthermore, IR can heighten mood disorders, including depression, anxiety, and reduced stress resilience [8].

Low-carbohydrate diets with high fat content have become popular for managing IR state comorbidities, such as prediabetes and type II diabetes (T2DM), and for preventing dyslipidemia [9,10]. A low-carbohydrate, high-fat (LCHF) diet improves insulin sensitivity and treats the IR state by improving systemic inflammation and enhancement of insulin signaling peripherally at the level of the iliac, visceral fat, and hepatic tissues [11] and centrally at the level of the brain tissue [12] and by ameliorating mitochondrial dysfunction and inhibiting apoptosis [13].

Accordingly, the current study aimed to investigate the gene expression of the brain IR and apoptosis markers in a drug-induced animal model of metabolic syndrome (MetS) and the possible preventive effects of LCHF. Our hypothesis suggested that the LCHF diet may offer protective effects against the neurological consequences of MetS. Moreover, a parallel group of rats fed with a conventional high-carbohydrate, low-fat diet (HCLF) was used to confirm the potential role of LCHF.

## 2. Materials and Method

This study was approved by the Biomedical Ethics Research Committee, Faculty of Medicine, King Abdulaziz University, Jeddah, Saudi Arabia (Reference No 33-21), and by the Animal Care and Use Committee (ACUC) at King Fahd Medical Research Center, King Abdul-Aziz University, Jeddah (Protocol Number: ACUC-20-09-21).

### 2.1. Animals and Experimental Protocol

This study was a nested study derived from the original research project by Alnami et al. [14]. Forty male Sprague-Dawley rats, weighing approximately 120 ± 20 g, were sourced from the Animal House at King Fahd Medical Research Center in Jeddah, Saudi Arabia. These rats were accommodated in metal mesh cages under conditions of 24 °C ± 3 °C in temperature, 40–70% humidity, and a 12/12-h light/dark cycle. For the initial 10 days, the rats were provided with unrestricted access to a standard laboratory chow diet and water, allowing them to adapt to their new surroundings. They were maintained under these conditions until they reached eight weeks of age.

**Figure 1:**
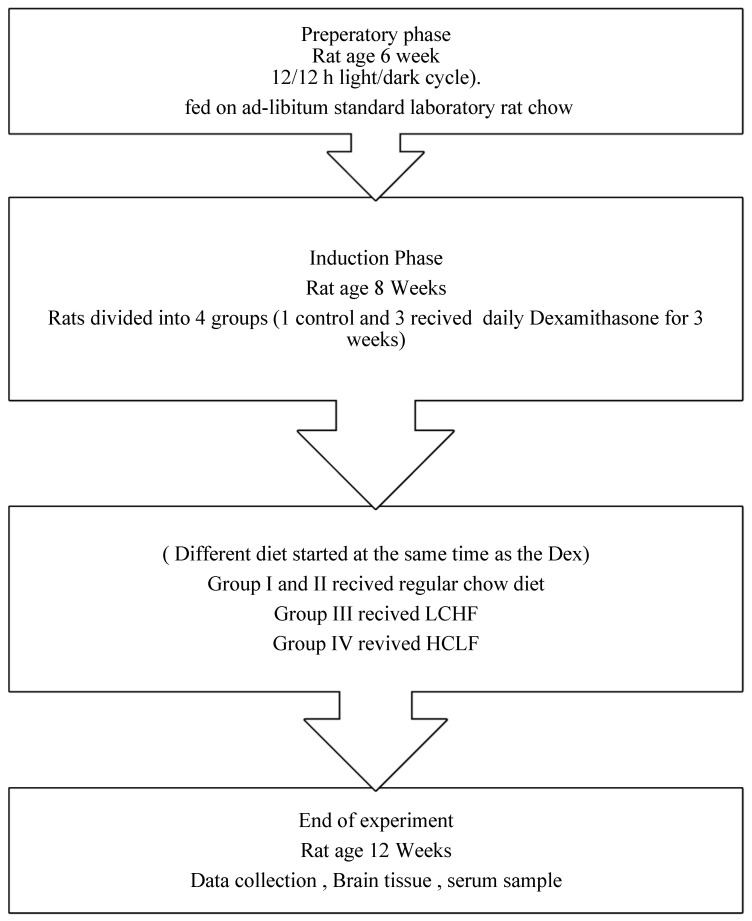
Flow chart of the experimental design.

The rats were grouped into four groups (10 rats/group) (Figure 1). Group I (Control) received a regular rat’s diet and was injected with (100 µL) saline subcutaneously. Groups II–IV were injected with (100 µL) dexamethasone (DEX) [14,15]. Group II (DEX): continued the chow diet. Group III (DEX + LCHF) rats were fed a LCHF comprising 85 kcl% fat (5.5 g/rat), 5 kcl% carbohydrates (1.0 g/rat), and 10 kcl% protein (2.0 g/rat). Group IV (DEX + HCLF): rats were fed a HCLF containing 85 kcl% carbohydrates (12.0 g/rat), 10 kcl% fat (2.0 g/rat), and 5 kcl% protein (1.0 g/rat). All diets used were isocaloric (60–61 calories per rat). The diet composition and preparation were previously described in detail by Alnami et al. [14,16].

### 2.2. Induction of Metabolic Syndrome

MetS was induced by dexamethasone injection (Saudi Pharmaceutical Industries (SPI), Riyadh, Saudi Arabia). A daily subcutaneously injection (250 µg/kg) was administered for four weeks, according to the previously described method [14,15].

### 2.3. Anthropometric Measurement and Tissue Sampling

Anthropometric measurements were conducted to evaluate the overall progress of the disease and the effectiveness of treatments. BMI was calculated based on the weekly measurements of height and body weight [17]. All animals were sacrificed, and a craniotomy was performed to dissect the intact brains. The brain volume (in mm^3^) was manually measurement using volumetric analysis; the brain volume was manually outlined using ImageJ image analysis software (http://rsbweb.nih.gov/ij/), referring to a standard rat brain atlas. The brain weight (in grams) was estimated according to the method described in [16]. After brain tissue dissection and isolation of the cerebral cortex, one cerebral cortex hemisphere of each was snap-frozen in liquid nitrogen and used immediately for RNA extraction. The other hemisphere was used for histological and immunohistochemical examinations.

### 2.4. Biochemical Investigations

At the conclusion of the four-week study, the rats were anesthetized using ether. Blood samples were withdrawn into plain plastic tubes from the retro-orbital venous plexus after 12 h of fasting. These samples were centrifuged for 20 min and then divided into aliquots and stored at −80 °C for later biochemical analysis. ELISA tests were carried out to determine fasting glucose (using a kit from Intertek, London, UK, CS605) and insulin levels (using the Rat Insulin ELISA kit from Novus Biologicals, LLC 10730, Centennial, Toronto, ON, USA), following the manufacturers’ guidelines. The level of insulin resistance (IR) was assessed using the HOMA-IR method, introduced by Matthews et al. This index was calculated by factoring in fasting insulin and blood glucose measurements: fasting insulin (μU/mL) × fasting plasma glucose (mg/dL) × 0.0551/22.5 [18].

Quantitative determination of serum IGF-1 (Rat IGF-1 ELISA Kit-ab213902), IGFBP-2 (Rat IGFBP-2 ELISA Kit-#ab207615; Abcam, Cambridge, MA, USA), IGFBP-5 (IGFBP5 (Rat) ELISA Kit-Catalog Number KA4319; Abnova, USA), and TNF-α (Rat TNF alpha ELISA Kit-ab100785; Abcam, Cambridge, MA, USA) was performed using sandwich-ELISA according to the manufacturers’ instructions.

### 2.5. Histological Study

A histological study was performed to ascertain the cellular and tissue level changes caused by MetS and the LCHF diet. Samples from the cerebral cortex were preserved in Bouin’s solution. Subsequently, the specimens underwent dehydration through a graded alcohol series, were clarified in xylene twice, and then were encased in molten paraffin. Using a rotary microtome, 5-micron thick slices were prepared and set on pristine slides. To facilitate histological analysis, these slices were then stained with hematoxylin and eosin (H&E), according to Bancroft and Layton (2019) [19].

### 2.6. Immunohistochemical (IHC) Study

An immunohistochemical study was performed to visualize and quantify specific protein expression levels related to inflammation, apoptosis, or other relevant pathways. For immunohistochemical detection of caspase-3 and S100B reactions, sections from the cerebrum were placed on positive slides and immune-stained using a standard avidin–biotin peroxidase complex system according to the kit used, followed by diaminobenzidine visualization [20]. Sections were treated with primary monoclonal antibodies for caspase-3 (Invitrogen, Carlsbad, CA, USA) at a 1:100 dilution in 3% BSA-PBS and were kept in a humidified chamber at 4 °C overnight. For the S100B antigen, sections underwent a heat treatment in 10 mM sodium citrate buffer in a microwave for 20 min, followed by cooling with deionized water. These samples were then exposed overnight at 4 °C to primary antibodies specific for S100 rabbit polyclonal antibody (DAKO, Glostrup, Denmark). The staining was revealed using biotin-tagged anti-rabbit secondary antibodies and streptavidin linked with horseradish peroxidase (DAKO Cytomation, Santa Clara, CA, USA), as guided by the manufacturer. The tissue sections were then stained with hematoxylin, dehydrated through ethanol and xylene steps, and prepared for mounting. PBS in place of the primary antibody served as a negative control, and any successful reaction was seen as a brown stain, either cytoplasmic or nuclear.

### 2.7. Morphometric Study

The area percentages of caspase-3 and S100B immunoreactions were measured using five immune-stained slides of five different rats for each group. Images of immunostained cells were transferred for analysis using VideoTest Morphology software, Version 4.0 (Saint Petersburg, Russia) with a built-in routine for area percentage measurement.

### 2.8. Molecular Studies

The protocol for RNA extraction was described previously by Bima et al. [21]. Briefly, RNA was isolated from 25- to 35-mg sections of the rats’ cerebral cortex using Tri-FastTM reagent, triazole, and chloroform (PeqLab. Biotechnologie GmbH, Nordhausen, Germany). The RNA quantity and quality were assessed using the NanoDropTM 2000 Spectrophotometer (Thermo Scientific, Waltham, MA, USA) following the manufacturer’s recommendations [21].

For cDNA synthesis, around 250 ng of total RNA underwent reverse transcription using the RevertAid First Strand cDNA Synthesis Kit (Thermo Scientific, USA, K1622). A thermal cycler (Techne TC-312, Staffordshire, UK) device was adjusted to the following program: 25 °C for 5 min (primer annealing), 42 °C for 60 min (reverse transcription), and a final increase to 70 °C for 5 min for inactivation and reaction termination.

The mRNA levels in the cerebral tissue were then assessed by RT-PCR on the Applied Biosystem 7500 detection system, utilizing the SYBR^®^ Green PCR Master Mix (Life Technology, Gaithersburg, MD, USA). The RCR reaction volume was 20 μL; 10 μL SYBR^®^ Green PCR Master Mix, 2 μL cDNA template, and 2 μL (10 pmol/μL) specific gene primers and was completed with 6 μL nuclease-free water. The RT-PCR amplification process involved an initial enzyme activation at 95 °C for 5 min, followed by 40 cycles that included template denaturation at 95 °C for 15 s and annealing/extension at 60 °C for 1 min [21]. The rats’ primer sequences used are presented in Table A1. GAPDH was used as an internal control (reference gene) to normalize the expression of the analyzed genes. The specificity of the primer sequences was checked using the Primer-BLAST program (https://www.ncbi.nlm.nih.gov/tools/primer-blast, accessed on 30 September 2021). Melting curve analysis was performed to check the specificity of amplified PCR products.

Relative gene expression levels were represented as ∆Ct = Ct target gene—Ct reference gene. Fold change in gene expression was calculated using comparative ∆∆CT method.

### 2.9. Statistical Analysis

Data were analyzed using GraphPad Prism software, version 9.5.1. The paired *t*-test was used to analyze weight, height, and BMI throughout the experiment. Intergroup differences were compared with ANOVA, and pairwise comparisons were carried out using the Tukey method. Pearson’s r correlation coefficient was computed to assess the linear relationships between different parameters. Data are presented as mean and standard deviation (SD). A *p*-value < 0.05 was considered statistically significant.

## 3. Results

### 3.1. Effect of LCHF and HCLF Diet on the Anthropometric Parameters and HOMA-IR

This study is part of a large project; some general data, such as anthropometric parameters and HOMA-IR data, were described previously by Alnami et al. [14]. In short, the body mass index (BMI) showed a significant increase in the DEX group compared to the control group. The LCHF diet caused a significant decrease in BMI compared to the DEX group on a regular diet. On the other hand, HCLF diet-fed rats showed a significant increase in BMI. Regarding the total brain weight and volume, a significant reduction was detected in the DEX compared to the control group, with a significant increase in the LCHF diet compared to the DEX group. At the same time, the HCLF diet-fed rats showed a significant decrease. Development of IR state was noted in DEX-treated groups of animals based on the significant increase in HOMA-IR compared to the control group. HOMA-IR showed a significant decrease in the LCHF diet group compared to the DEX group. On the other hand, HCLF diet-fed rats showed a significant increase in HOMA-IR compared to the negative control and LCHF diet-fed rat groups (Table 1). These data indicated a protective role for the LCHF diet against developing IR associated with MetS.

### 3.2. Effect of LCHF & HCLF Diet on Brain Histology

**Figure 2:**
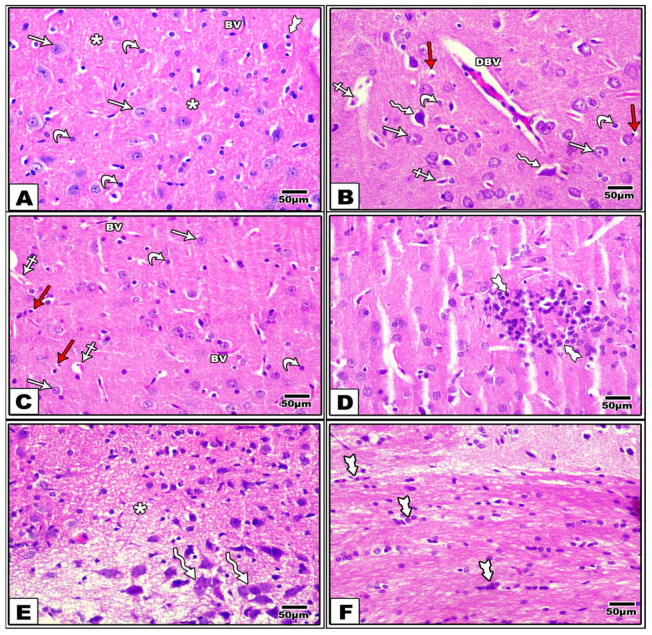
A photomicrograph of sections of the cerebral cortex stained with H&E. (**A**) Controls showing large pyramidal cells (arrows) with long apical dendrites, vesicular nuclei, and basophilic cytoplasm, as well as rounded granular cells (curved arrows) with large rounded vesicular nuclei. The nuclei of neuroglial cells (tailed arrow) and normal blood vessels (BV) are seen in the neuropil (*). (**B**) (Dex) shows necrotic cells (crossed arrows) with acidophilic cytoplasm, apoptotic cells (red arrows) with pyknotic nuclei, degenerated pyramidal cells (zigzag arrows) with darkly stained nuclei and perineural spaces, and dilated congested blood vessels (DBVs) with perivascular spaces. Notice the presence of some normal pyramidal cells (arrows) and granular cells (curved arrows). (**C**) (Dex + LCHF) shows multiple normal pyramidal cells (arrow) and granular cells (curved arrow), and normal blood vessels (BVs) in the neuropil. Few necrotic cells (crossed arrows) and apoptotic cells (red arrows) are seen. (**D**–**F**) (Dex + HCLF). (**D**) shows a collection of neuroglial cells (tailed arrows). (**E**) shows degenerated pyramidal cells (zigzag arrows) with darkly stained pyknotic nuclei and cytoplasmic vacuolation with hypercellularity and vacuolation of neuropil (*). (**F**) shows gliosis.

Examination of H&E-stained sections from the control group showed that the cerebral cortex was formed of pyramidal, granular, and neuroglial cells. The pyramidal cells were large in size with long apical dendrites, vesicular nuclei, and basophilic cytoplasm. The granular cells appeared rounded in shape with large, rounded vesicular nuclei. Neuroglial cells appeared smaller in size with small, deeply stained nuclei. The homogenous eosinophilic background (neuropil) between the nerve cells was formed of neuronal and neuroglial cell processes and contained normal blood vessels (Figure 2A). On the other hand, the DEX-treated group showed distinct histological changes in the form of necrotic cells with acidophilic cytoplasm and apoptotic cells with pyknotic nuclei. Some pyramidal cells were degenerated and irregularly shaped with darkly stained nuclei and perineural spaces. Some regions of the neuropil showed dilated congested blood vessels with perivascular spaces (Figure 2B). Light microscopic examination of cortical regions of the DEX + LCHF group showed brain tissue was a morphology similar to normal. Multiple pyramidal and granular cells appeared normal; however, few necrotic and apoptotic cells were found. The blood vessels in the neuropil appeared normal (Figure 2C). Last, the DEX + HCLF group revealed numerous pathological changes. There was a collection of neuroglial cells (Figure 2D). Multiple degenerated pyramidal cells with darkly stained pyknotic nuclei and cytoplasmic, vacuolation together with hypercellularity and vacuolation of neuropil, were demonstrated (Figure 2E), as well as gliosis (Figure 2F).

### 3.3. Effect of LCHF and HCLF Diet on the Expression of IGF-1, IGF-1R, IGFBP-2, and IGFBP-5 Expression in DEX-Treated Rats

**Figure 3:**
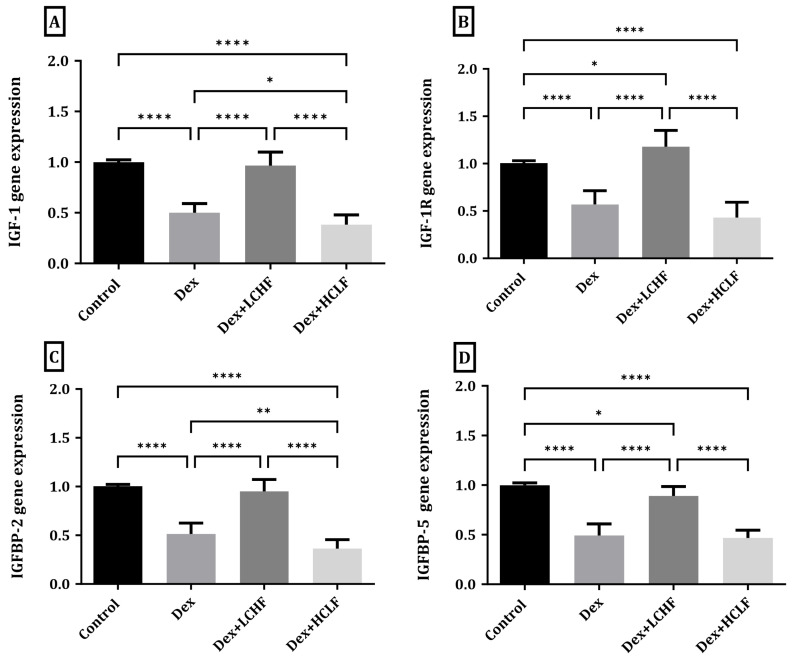
Low-carbohydrate, high-fat and high-carbohydrate, low-fat diets impact the brain’s IGF and IGF-binding protein gene expression. (**A**) IGF-1 gene expression; (**B**) IGF-1 R gene expression; (**C**) IGFBP-2 gene expression; (**D**) IGFBP-5 gene expression Data were expressed as the mean ± SD. DEX, DEX + LCHF, and DEX + HCLF groups. DEX: rats treated with dexamethasone to induce MetS; DEX + LCHF: rats treated with dexamethasone to induce MetS and fed the low-carbohydrate, high-fat diet; DEX + HCLF: rats treated with dexamethasone to induce MetS and fed the high-carbohydrate, low-fat diet (applies to all figures). Y axis (Fold expression relative to control). Statistically significant if *p* ≤ 0.05 *; *p* < 0.01 **; *p* < 0.0001 ****. IGF-1: Insulin growth factor-1, IGF-1 R: Insulin growth factor-1 receptors, IGFBP-2: Insulin growth factor binding protein-1, and IGFBP-5: Insulin growth factor binding protein-5.

**Figure 4:**
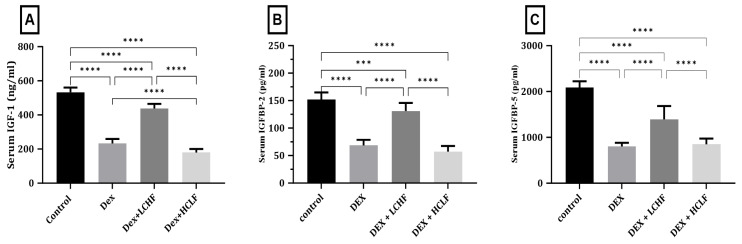
The impact of low-carbohydrate, high-fat and high-carbohydrate, low-fat diets on IGF and IGF-binding proteins serum level. (**A**) IGF-1 serum level; (**B**) IGFBP-2 serum level; (**C**) IGFBP-5 serum level. Data are expressed as mean ± SD. Statistically significant if *p* < 0.001 ***; *p* < 0.0001 ****. IGF-1: Insulin growth factor-1, IGFBP-2: Insulin growth factor binding protein-2, and IGFBP-5: Insulin growth factor binding protein-5.

As shown in Figure 3, DEX treatment resulted in a significant (*p* < 0.0001) decrease in both IGF-1 and IGF-1R expression compared to the control group. Rats fed the HCLF diet showed further exaggeration with significantly decreased IGF-1 compared to the control (*p* < 0.0001) and Dex groups (*p* < 0.05); however, this change did not reach significant levels in IGF-1R compared to DEX-treated rats. DEX + LCHF, on the other hand, led to a significant increase in both IGF-1(*p* < 0.0001) and IGF-1R (*p* < 0.0001) compared to the DEX-treated rats. Moreover, the expression of IGFBP-2 and IGFBP-5 decreased significantly (*p* < 0.0001) in the DEX-treated group versus the control rats. At the same time, the HCLF diet significantly reduced the expression of IGFBP-2 (*p* < 0.05), while no change was observed in IGFBP-5 compared to the DEX-treated group. In contrast, the LCHF diet increased the expression of IGFBP-2 and IGFBP-5 significantly (*p* < 0.0001) compared to the DEX-treated group. The changes in gene expression were similar to the changes observed in serum (Figure 4).

### 3.4. Effect of LCHF and HCLF Diet on the Expression of S100B, TNF-α, and BDNF in DEX-Treated Rats

**Figure 5:**
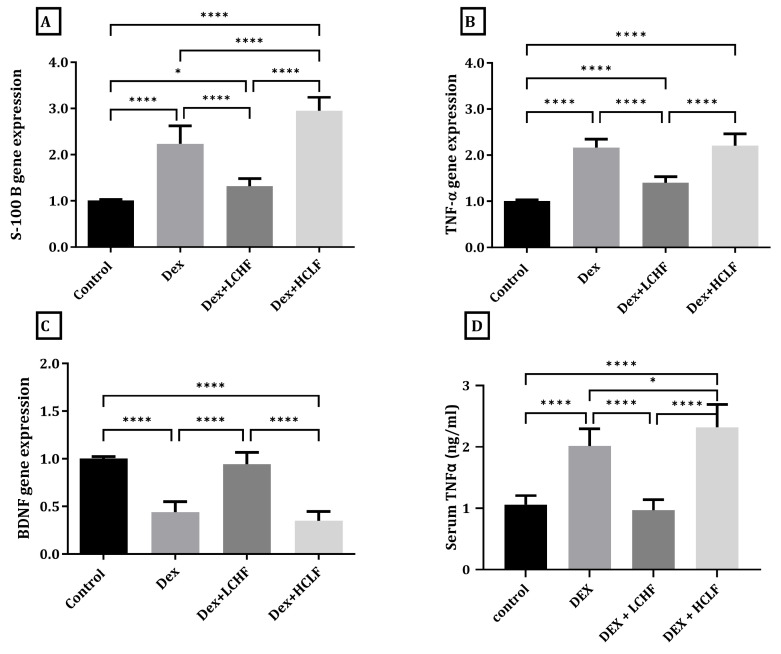
Low-carbohydrate, high-fat and high-carbohydrate low-fat diets impact the brain’s S100B, TNF-α, and BDNF gene expression. (**A**) S100B gene expression; (**B**) TNF-α gene expression; and (**C**) BDNF gene expression. (**D**) TNF-α serum level. Y axis of gene expression (gold expression relative to control). Data were expressed as mean ±SD. DEX, DEX + LCHF, and DEX + HCLF groups. Statistically significant if *p* ≤ 0.05 *; *p* < 0.0001 ****. TNF-α: Tumor necrosis factor-alpha, BDNF: Brain-derived neurotrophic factor.

As depicted in Figure 5, in comparison to the control group, the DEX treated group showed a significant increase in the cortical expression of S100B and TNF-α (*p* < 0.0001) and a significant decrease in BDNF (*p* < 0.0001). Moreover, DEX rats fed the HCLF diet had significantly increased S100B (*p* < 0.0001) compared to the DEX group with an insignificant change in BDNF cortical expression. On the other hand, feeding rats the LCHF diet significantly (*p* < 0.0001) decreased the expression of S100B and TNF-α compared to DEX-treated rats with a significant increase in the expression of BDNF (*p* < 0.0001). Furthermore, serum TNF-α showed a significant increase in the DEX and DEX + HCLF groups compared to the control group, while the DEX + LCHF group showed a level similar to the control group. Interestingly, a significant, inverse correlation (r= −0.8668, *p* < 0.0001) existed between BDNF and TNF-α.

### 3.5. Effect of LCHF and HCLF Diet on the Expression of Caspase-3, Bax, and Bcl-2 Expression in DEX-Treated Rats

**Figure 6:**
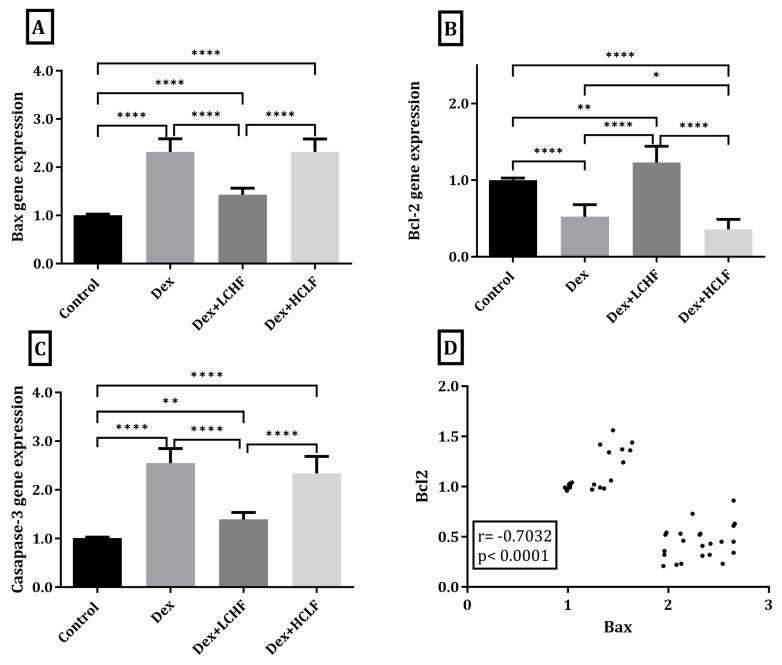
The impact of low-carbohydrate, high-fat and high-carbohydrate, low-fat diets on the brain’s Bax, Bcl-2, and caspase-3 gene expression. (**A**) Bax B gene expression; (**B**) Bcl-2 gene expression; (**C**) caspase-3 gene expression; and (**D**) Pearson’s correlation between Bcl-2 and Bax. Y axis (fold expression relative to control). Data are expressed as mean ± SD. DEX, DEX + LCHF, and DEX + HCLF groups. Statistically significant if *p* ≤ 0.05 *; *p* < 0.01 **; *p* < 0.0001 ****.

As illustrated in Figure 6, in DEX-treated rats, there was a significant increase in the cortical expression of caspase-3 and Bax compared to the control group (*p* < 0.0001), while there was a significant decrease in Bcl-2 versus the control rats (*p* < 0.0001). No significant change was observed in Bax or caspase-3 levels in the HCLF diet group compared to the DEX group. However, a significant decrease in Bcl-2 cortical expression was observed (*p* < 0.05). Rats fed the LCHF diet had significantly decreased expression of caspase-3 and Bax compared to DEX-treated rats (*p* < 0.0001) and showed a significant increase in the expression of Bcl-2 (*p* < 0.0001). Furthermore, there was a significant inverse correlation (r = −0.7032, *p* < 0.0001) between Bax and Bcl-2.

### 3.6. Effect of LCHF and HCLF Diet on Brain Caspase-3 and S100B Expression

**Figure 7:**
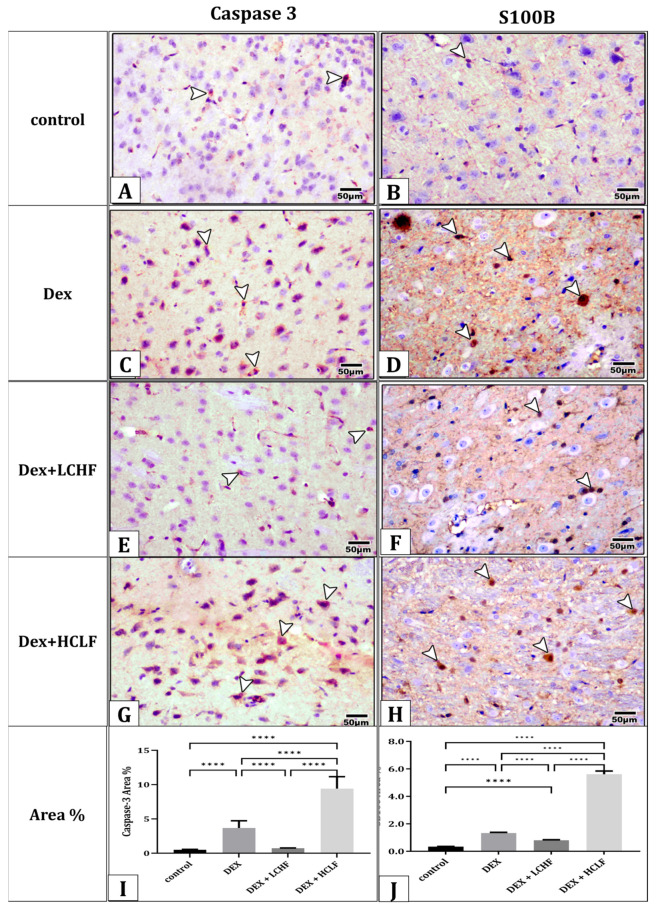
A photomicrograph of sections of the cerebral cortex immunohistochemically stained with caspase-3 and S100B. (**A**) Control group showing minimal caspase-3 expression (arrowheads). (**B**) Control group showing minimal S100B expression (arrowheads). (**C**) DEX group showing moderate caspase-3 reaction (arrowheads). (**D**) DEX group showing moderate S100B reaction (arrowheads). (**E**) DEX + LCHF group showing near control caspase-3 expression. (**F**) DEX + LCHF group showing near control S100B expression. (**G**) DEX + HCLF group showing strong caspase-3 expression. (**H**) DEX + HCLF group showing strong S100B expression. (**I**,**J**) represent the mean area % of caspase-3 and S100B expression, respectively. Data are expressed as mean ± SD and on the high-carbohydrate, low-fat diet. Statistically significant if *p* < 0.0001 ****.

Immunohistochemistry was performed to study the expression of caspase-3 and S100B in brain cells (Figure 7). Minimal caspase-3 and S100B-positive cells could be observed in the immunohistochemically stained sections of the control group. Numerous caspase-3- and S100B-positive cells could be observed in the immunostained sections of the DEX group. On the other hand, the DEX + LCHF group showed a distribution pattern of the positively immune-stained neuronal cells by caspase-3 and S100B near that of the controls. In the DEX + HCLF group, the distribution of positively immunohistochemically neuronal cells stained for caspase-3 and S100B was higher than in the DEX group. The mean area % of caspase-3 and S100B reaction in all groups is presented in Figure 7I,J. There was a significant increase (*p* ≤ 0.001) in caspase-3 and S100B reactions in the DEX vs. DEX + HCLF group compared with the control group but insignificant increases in DEX + LCHF compared to the control group, all of which indicated the protective role of LCHF against apoptosis and neuronal cells stress in this model.

### 3.7. Correlations of All Measured and Calculated Data in All Studied Rats

**Figure 8:**
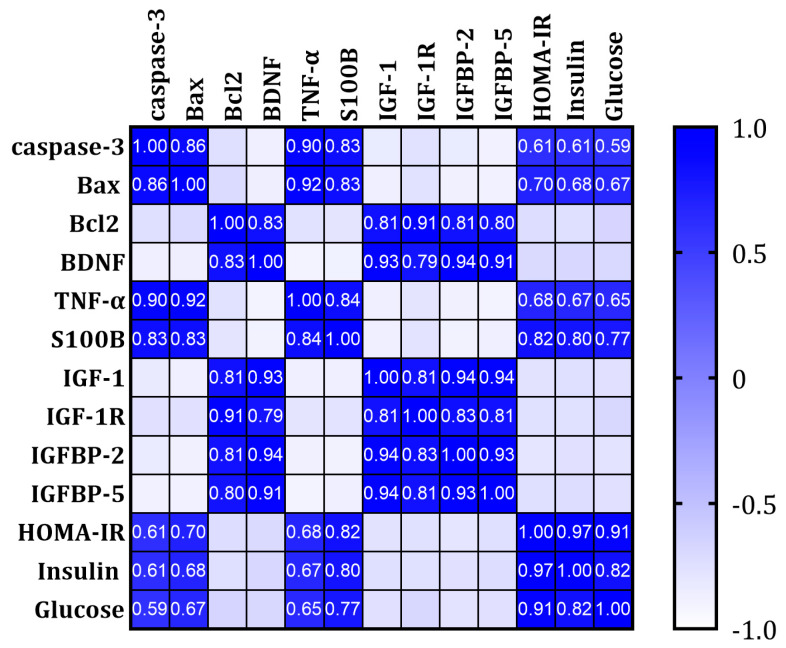
Pearson’s correlations of all measured and calculated data in all studied rats merged.

Significant correlations were detected between the measured and the calculated parameters in all rats merged, as shown in Figure 8. HOMA-IR, insulin, and glucose levels were compared to IR, apoptosis, inflammatory, and neurogenesis parameters. All brain IR markers (IGF-1, IGF-1, IGFBP-2, IGFBP-5) were strongly, negatively correlated with HOMA-IR. The apoptotic markers caspase-3 and Bax showed a strong, positive correlation with high glucose, HOMA-IR, and inflammatory markers (TNF-α), while the anti-apoptotic Bcl-2 expression was negatively correlated. Markers of brain inflammation or brain damage (S100B and TNF-α) also showed a strong, positive correlation with insulin and HOMA-IR; in contrast, brain neurogenesis and neuroprotection markers (BDNF) showed a strong, negative correlation. All correlations were significant (*p* < 0.0001).

## 4. Discussion

The current study revealed that MetS is associated with changes in the brain at the morphological and molecular levels.

Our study confirmed the development of brain IR in a DEX-treated rat model of MetS by decreasing the cerebral expression of IGF-1, IGF-1R, IGFBP-2, and IGFBP-5. Furthermore, the study showed the protective role of LCHF diet in this model.

Aguirre et al. and de la Monte et al. reported a decrease in IGF-1/IGF-1R expression in a model of IR and streptozocin-induced T2DM, which agreed with our findings [22,23]. Inhibited IGF-1/IGF-1R in the brain is a marker of impaired brain energy metabolism and neurotransmitter function, as these markers mediate neuronal survival, plasticity, energy metabolism, and neurotransmitter function [24,25]. Moreover, IGF-1/IGF-1R expression inhibition is an essential mediator of brain IR and its consequent pathology [26,27].

Dysregulation of insulin and IGF-1 signaling could explain many associated brain pathological changes of MetS. Insulin is involved in oxidative stress, enhanced apoptosis, impaired protein synthesis, dysregulated autophagy, and neuroinflammation [28,29]. Thus, the interplay among hyperinsulinemia, insulin receptor resistance, downregulation of insulin-like growth factor-1 receptor, and IR signaling pathways could explain the molecular basis of the brain IR state [22,30].

IGF-binding proteins (IGFBP) are a family of six transporter proteins that depend on and follow the IGF function [31,32]. IGFBPs mediate the actions of IGFs by regulating their bioavailability. IGFBP-2 [32] and IGFBP-5 [33] are the key members contributing to unique physiological and metabolic processes. These metabolic processes include phosphatidylinositol 3-kinase (PI3K)/alpha serine/threonine-protein kinase (Akt) signaling and mitogen activated protein kinase (MAPK) signaling-mediated pathways, such as development, cell proliferation, differentiation, and migration, as well as inhibition of apoptosis. Moreover, they also include cyclin-dependent kinase inhibitor 1 (p21), as well as the angiogenic mediator vascular endothelial growth factor (VEGF) [32,33].

Hyperinsulinemia of MetS and the IR state downregulate the expression of IGF-binding proteins [34]. This finding was confirmed in the current study. Moreover, low expression levels of IGFBP-2 at the adipose tissue mRNA and its circulating protein levels are connected to hyperglycemia and hypertriglyceridemia and positively correlate with insulin sensitivity. Accordingly, low levels of IGFBP-2 at the gene expression level and low circulating protein are considered MetS biomarkers [35,36].

IGFBP-5 chromosomal deletion and low expression are associated with increased adiposity and marked glucose intolerance [37]. Its downregulation is strictly linked to the IR state [38]. Like in our results, IGFBP-5 mRNA and protein levels were decreased in a critical IR-related comorbidity: non-alcoholic fatty liver disease (NAFLD) [33]. This finding proved its involvement in the pathogenesis of IR syndrome.

This study revealed a significant increase in neuroinflammation, and brain tissue damage was confirmed by increased cerebral expression of S100B and TNF-α (differing in degree) in all groups that received dexamethasone, with decreased BDNF expression in DEX and DEX + HCLF only. In the DEX + LCHF group, although there was an elevation in the biomarkers, it was not as pronounced as in the other groups and was almost comparable to control group.

The human brain-derived neurotrophic factor (BDNF) is a master neurotrophin highly expressed in the hippocampus. It promotes neurogenesis, neuroplasticity, and neuroprotection. Furthermore, BDNF is an anorexigenic protein that regulates the body’s energy hemostasis [39]. Like in our results, Lee et al. [40], Motamedi et al. [41], Jamali et al. [42], and Abdulsada et al. [39] reported that BDNF is downregulated in MetS and is involved in the pathogenesis of neurodegeneration.

S100B is a calcium-binding protein involved in regulating brain metabolism. It is present in high concentrations in the glial cells of the central nervous system. Its expression is increased in the presence of brain tissue damage [43] and chronic cerebral ischemia, as its level is inversely proportional to cerebral blood flow. Moreover, oxidative stress [44] and neuroinflammation [45] induce its expression. These findings were confirmed in the current study, as there was concomitantly increased expression of cerebral TNF-α in rats with MetS compared to the controls in parallel with increased S100B. The serum level of S100B is directly correlated with hyperglycemia and hyperinsulinemia in streptozocin-induced type II diabetes as a model of IR [46]. This concept supports our hypothesis that IR is associated with increased expression of S100B in the brain. These mechanisms are proposed to explain the brain’s IR pathophysiology and development [47]. In this study, increased expression of cerebral S100B was also detected at the protein level, as revealed by immunohistochemical examination, following the same expression pattern at the molecular level.

Increased apoptosis is a hallmark of MetS. It was confirmed in the present study by the increased expression of caspase-3 and Bax apoptotic genes with the decrease in the Bcl-2 anti-apoptotic gene.

Increased cerebral apoptosis in MetS and IR in our study coincided with what was previously published by Nuzzo et al. [48], Rahmati et al. [49], and Feng et al. [50].

In physiological conditions, glucose is the primary energy source for the brain, and the continuous supply of glucose is compulsory for neuronal activities, including regulation of apoptosis, neurogenesis, and neuronal firing [51]. In MetS and other IR conditions, despite hyperglycemia, the brain experiences decreased glucose transport and utilization, leading to reduced glucose availability for brain cells [52] and dysregulation of neuronal apoptosis [53]. This mechanism could be an additive factor in developing brain IR and its neurodegenerative consequences.

Moreover, the brain energy supply is diminished due to microvascular and macrovascular changes and defective glucose utilization. This cerebral, energetic defect induces cerebral cell apoptosis [54].

In our study, the brain weight and volume were reduced in the MetS rat group compared to the normal control rats. This finding correlates with all of the molecular changes (neuroinflammation and apoptosis) and the high HOMA-IR value, indicating IR and its associated hyperinsulinemia [8,55,56,57]. Chronic hyperinsulinism [58] and atherosclerosis [57] are important mechanisms that explain this finding. Hyperinsulinemia increases peripheral lactate levels, subsequently increases lactate’s net flux across the blood–brain barrier (BBB), and decreases energy metabolism within the brain cells, especially the astrocytes, leading to brain atrophy [59]. Oxidative stress is an essential mechanism of IR [60]. It also leads to oxidation-mediated cell death, gliosis, and apoptosis, which could explain cerebral atrophic changes [61].

Reduced brain weight coincided with some histological changes, as our study showed evidence of apoptosis and degenerated neuroglial cells and pyramidal cells.

Neuroimaging studies revealed structural and functional changes in patients with chronic IR as documented in T2DM compared with healthy individuals [8]. Furthermore, cerebral atrophy is detected more frequently in patients with IR and T2DM than in those without these conditions at the same age [62].

Based on these previously discussed morphological, biochemical, and molecular effects of MetS and IR, we investigated the proposed protective effect of the LCHF diet in comparison to the regular or HCLF diet in the MetS rat model.

LCHF diet is also known as the ketogenic diet (KD), as it induces the production of small amounts of ketone bodies (KBs). This condition is called “nutritional ketosis,” as it does not affect the normal blood pH of 7.4, which is benign, physiological, and safe [63]. The KD affects IR-related disorders. It improves insulin sensitivity, decreases protein glycation, and has anti-inflammatory and antioxidant properties [64].

Increases in cerebral expression of IGF-1, IGF-1R, IGFBP-2, and IGFBP-5 in the LCHF-fed rat model compared to the MetS group were noted in the present study at the molecular and serum protein levels. These findings agree with Montella et al. and Lilamand et al. [65,66]. This effect could be explained by the brain’s metabolic shift to use ketones as a source of energy rather than carbohydrates [66].

Our study revealed a significant increase in BDNF with decreases in S100B and TNF-α expression in the LCHF-fed rats compared to the MetS group.

Previous reports have indicated increased BDNF under nutritional and therapeutic ketosis [67,68,69]. β-hydroxybutyrate (βOHB) is the leading enhancer of BDNF expression [70], as it protects the neurons against oxidative stress and enhances mitochondrial respiration [71].

In addition, a decrease in cerebrospinal fluid S100B in rats has been reported under the effects of the KD [72,73]. Like our study, Ziegler et al. (2004) [74] and Vizuete et al. (2013) [72] reported a decrease in S100B in response to the KD.

Moreover, the anti-neuroinflammatory effect of LCHF on brain cells, as evidenced by decreased TNF-α expression, was reported by Jiang et al. [75] in their review article and was confirmed in the current study. The KB-mediated anti-inflammatory effect is induced by the release of anti-inflammatory cytokines and inhibited pro-inflammatory types, such as NF-κB and Ikappa B kinase [76]. βOHB suppresses stress-related inflammasome production in the endoplasmic reticulum [77]. Moreover, the role of KD in neuroprotection is also mediated by increasing corticosterone levels, exaggerating autophagy and oligodendrocyte regeneration [64].

This study also showed a decrease in apoptotic gene expression of Bax and caspase-3 and a decrease in the immunoreactivity of caspase-3 with a simultaneous and correlated increase in the Bcl-2 anti-apoptotic gene. These findings confirm the anti-apoptotic effect of the ketogenic LCHF diet [12,13,78]. This effect is related and complementary to its anti-neuroinflammation [79], antioxidant [80], and mitochondrial enhancement effects [81,82].

In the current study, brain weight and volume and the cerebral cortex histological picture were close to normal in the MetS model fed the LCHF diet, indicating the protective role of this diet. This finding also corresponded to a decrease in the HOMA-IR value. Davis et al. [83] discussed the restoration of brain volume and metabolic activity under the effect of diet-induced ketosis in their review article. There is controversy about the impact of ketosis and its duration on brain volume and weight. Our results suggest that physiological ketosis preserves brain volume and weight. Furthermore, the antioxidative, mitochondrial biogenesis-enhancing, anti-inflammatory, and anti-apoptotic effects could explain the beneficial impact of a ketogenic diet on brain volume and weight [84,85].

To determine the impact of the LCHF diet and validate its protective influence on the studied variables, we also evaluated a parallel group of rats on a conventional high-carbohydrate, low-fat diet (HCLF). Notably, despite being isocaloric to the LCHF diet, the HCLF diet led to a significant decline in all examined markers related to cerebral IR, inflammation, and apoptosis. The development of hyperinsulinemia could be explained by the HCLF’s high carbohydrates and low-fat or even its high carbohydrates alone and the consequent effect resulting from high insulin, as described by Joseph et al. [86]. By further comparing the results of the HCLF-fed group of animals to those provided with LCHF, our hypothesis was better illustrated and confirmed.

The implications of the current study are reflective of understanding of brain insulin resistance (BIR). The expression changes in IGFBP-2 and IGFBP-5 could serve as potential indicators of BIR severity. These variations imply a connection between systemic and brain insulin resistance and might illuminate broader metabolic dysfunctions. By targeting these expressions, we could open new treatment avenues for BIR. Additionally, our observations on the altered levels of proteins, such as S100B, TNF-α, and BDNF, provide deeper insights into BIR’s molecular intricacies. Specifically, with S100B and TNF-α linked to neuroinflammation and a decline in BDNF signifying diminished neurotrophic support, we can better appreciate the hallmarks of BIR. The current study also underscored the potential of the low-carb, high-fat (LCHF) diet in BIR prevention and treatment. However, tailored approaches are necessary, and we emphasize the need for continued research in this area.

## 5. Conclusions

The current study results concluded that brain IR is a fundamental element of MetS. MetS is associated with morphological changes due to decreased brain weight and volume and molecular changes in the form of dysregulated gene expression of BDNF, S100B, and TNF-α. In addition, there is a disturbed insulin growth factor-1 signaling pathway, as presented by inhibited gene expression of insulin-like growth factor 1/insulin growth factor receptor (IGF-1/IGF-1R), IGFBP-2, and IGFBP-5. Increased apoptosis is a hallmark of MetS, as presented by the increased expression of caspase-3 and Bax apoptotic genes with a simultaneous decrease in the Bcl-2 anti-apoptotic gene. The administration of a LCHF has a protective effect on preventing the metabolic effects of MetS. This effect was proven at the biochemical and molecular levels, as presented by restoring the biochemical and molecular parameters nearly to those of the control group expression levels. Further molecular research is recommended to explore the molecular mechanism of the ameliorative effect of the LCHF diet on MetS-associated cerebral IR.

## Figures and Tables

**Table 1:** All studied animal groups’ anthropometric parameters and HOMA-IR.

	Control	DEX	DEX + LCHF	DEX + HCLF	*p*-Value (ANOVA)
BMI (baseline) [14]	0.51 ± 0.031	0.52 ± 0.05	0.53 ± 0.05	0.51 ± 0.044	0.87
BMI (Dex induction) [14]	0.65 ± 0.07	0.76 ± 0.08 ^a^	0.51 ± 0.034 ^a,b^	0.93 ± 0.041 ^a,b,c^	<0.001
BMI (post-stopping Dex) [14]	0.72 ± 0.05	0.88 ± 0.05 ^a^	0.53 ± 0.06 ^a,b^	0.97 ± 0.07 ^a,c^	<0.001
Brain weight (g) (post stopping Dex)	3.89 ± 0.21	2.83 ± 0.19 ^a^	3.76 ± 0.43 ^b^	2.56 ± 0.17 ^a,c^	<0.001
Brain volume (mm^3^) (post-stopping Dex)	1.81 ± 0.11	1.52 ± 0.09 ^a^	1.76 ± 0.06 ^b^	1.44 ± 0.13 ^a,c^	<0.001
HOMA-IR [14]	2.43 ± 0.19	4.89 ± 0.39 ^a^	2.67 ± 0.29 ^b^	5.76 ± 0.94 ^a,b,c^	<0.001

Data are presented as mean ± SD. SD: standard deviation. Statistically significant if *p* ≤ 0.05. ^a^: Statistically significant compared to the control group. ^b^: Statistically significant compared to the DEX group. ^c^: Statistically significant compared to the DEX + LCHF group. Statistical analysis was performed using one-way ANOVA followed by Tukey’s post hoc test. BMI and HOMA-IR data are from a previous study by Alnami et al. [14].

## Data Availability

The data presented in this study are available on request from the corresponding author.

## Appendix A

**Table A1:** The primer sequences of the analyzed genes.

Gene		The Primer Sequence	Reference
*S100B*	forward	5′-TAG TCC TTG GAC ACC GAA GC-3′	[87]
	reverse	5′-CAT CAA TGA GGG CAA CCA T-3′	
*BDNF*	forward	5′-CAG GGG CAT AGA CAA AAG-3′	[88]
	reverse	5′-CTT CCC CTT TTA ATG GTC-3′	
*TNF-α*	forward	5′-AAT GGC CTC CCT CAT CAG TT-3′	[89]
	reverse	5′-CCA CTT GGT GGT TTG CTA CGA-3′	
*IGF-1*	forward	5′-AAC CTG CAA AAC ATC GGA AC-3′	[90]
	reverse	5′-GCA GCC AAA ATT CAG AGA GG-3′	
*IGF-1 R*	forward	5′-GAC AGT GAA TGA GGC TGC AA-3′	[90]
	reverse	5′-CCA GCC ATC TGG ATC ATC TT-3′	
*IGFBP-2*	forward	5′-AGA ATG ATG GGG CTC ACG C-3′	[91]
	reverse	5′-TTT CTG CTG GTG TTT GGG GT-3′	
*IGFBP-5*	forward	5′-GGG CTC TTT CGT GCA TTG TG-3′	[91]
	reverse	5′-ATC TTG GTT TGC TCG CCG TA-3′	
*Bax*	forward	5′-CGG CGA ATT GGA GAT GAA CTG G-3′	[92]
	reverse	5′-CTA GCA AAG TAG AAG AGG GCA ACC-3′	
*Bcl-2*	forward	5′-TGT GGA TGA CTG ACT ACC TGA ACC-3′	[93]
	reverse	5′-CAG CCA GGA GAA ATC AAA CAG AGG-3′	
*Caspase-3*	forward	5′-GTG GAA CTG ACG ATG ATA TGG C-3′	[93]
	reverse	5′-CGC AAA GTG ACT GGA TGA ACC-3′	
*GAPDH*	forward	5′-TCC CTC AAG ATT GTC AGC AA-3′	[89]
	reverse	5′-AGA TCC ACA ACG GAT ACA TT-3′	

## References

[1] Raichle M.E., Gusnard D.A. (2002). Appraising the brain’s energy budget. Proc. Natl. Acad. Sci. USA.

[2] Milstein J.L., Ferris H.A. (2021). The brain as an insulin-sensitive metabolic organ. Mol. Metab..

[3] Wrigley S., Arafa D., Tropea D. (2017). Insulin-Like Growth Factor 1: At the Crossroads of Brain Development and Aging. Front. Cell Neurosci..

[4] Csajbók É.A., Tamás G. (2016). Cerebral cortex: A target and source of insulin?. Diabetologia.

[5] Gray S.M., Barrett E.J. (2018). Insulin transport into the brain. Am. J. Physiol. Cell Physiol..

[6] Agrawal R., Reno C.M., Sharma S., Christensen C., Huang Y., Fisher S.J. (2021). Insulin action in the brain regulates both central and peripheral functions. Am. J. Physiol. Endocrinol. Metab..

[7] Goldstein B.J. (2002). Insulin resistance as the core defect in type 2 diabetes mellitus. Am. J. Cardiol..

[8] Arnold S.E., Arvanitakis Z., Macauley-Rambach S.L., Koenig A.M., Wang H.-Y., Ahima R.S., Craft S., Gandy S., Buettner C., Stoeckel L.E. (2018). Brain insulin resistance in type 2 diabetes and Alzheimer disease: Concepts and conundrums. Nat. Rev. Neurol..

[9] Evert A.B., Dennison M., Gardner C.D., Garvey W.T., Lau K.H.K., MacLeod J., Mitri J., Pereira R.F., Rawlings K., Robinson S. (2019). Nutrition Therapy for Adults With Diabetes or Prediabetes: A Consensus Report. Diabetes Care.

[10] Ebbeling C.B., Knapp A., Johnson A., Wong J.M.W., Greco K.F., Ma C., Mora S., Ludwig D.S. (2022). Effects of a low-carbohydrate diet on insulin-resistant dyslipoproteinemia-a randomized controlled feeding trial. Am. J. Clin. Nutr..

[11] Lee S., Keirsey K.I., Kirkland R., Grunewald Z.I., Fischer J.G., de La Serre C.B. (2018). Blueberry Supplementation Influences the Gut Microbiota, Inflammation, and Insulin Resistance in High-Fat-Diet-Fed Rats. J. Nutr..

[12] Shippy D.C., Wilhelm C., Viharkumar P.A., Raife T.J., Ulland T.K. (2020). β-Hydroxybutyrate inhibits inflammasome activation to attenuate Alzheimer’s disease pathology. J. Neuroinflamm..

[13] Guo Y., Zhang C., Shang F.-F., Luo M., You Y., Zhai Q., Xia Y., Suxin L. (2020). Ketogenic Diet Ameliorates Cardiac Dysfunction via Balancing Mitochondrial Dynamics and Inhibiting Apoptosis in Type 2 Diabetic Mice. Aging Dis..

[14] Alnami A., Bima A., Alamoudi A., Eldakhakhny B., Sakr H., Elsamanoudy A. (2022). Modulation of Dyslipidemia Markers Apo B/Apo A and Triglycerides/HDL-Cholesterol Ratios by Low-Carbohydrate High-Fat Diet in a Rat Model of Metabolic Syndrome. Nutrients.

[15] Sivabalan S., Renuka S., Menon V.P. (2008). Fat feeding potentiates the diabetogenic effect of dexamethasone in Wistar rats. Int. Arch. Med..

[16] Ble-Castillo J.L., Aparicio-Trapala M.A., Juárez-Rojop I.E., Torres-Lopez J.E., Mendez J.D., Aguilar-Mariscal H., Olvera-Hernández V., Palma-Cordova L.C., Diaz-Zagoya J.C. (2012). Differential effects of high-carbohydrate and high-fat diet composition on metabolic control and insulin resistance in normal rats. Int. J. Environ. Res. Public Health.

[17] Novelli E.L.B., Diniz Y.S., Galhardi C.M., Ebaid G.M.X., Rodrigues H.G., Mani F., Fernandes A.A.H., Cicogna A.C., Novelli Filho J.L.V.B. (2007). Anthropometrical parameters and markers of obesity in rats. Lab. Anim..

[18] Matthews D.R., Hosker J.P., Rudenski A.S., Naylor B.A., Treacher D.F., Turner R.C. (1985). Homeostasis model assessment: Insulin resistance and β-cell function from fasting plasma glucose and insulin concentrations in man. Diabetologia.

[19] Suvarna S.K., Layton C., Bancroft J.D. (2019). Bancroft’s Theory and Practice of Histological Techniques.

[20] Jackson P., Blythe D., Suvarna S.K., Layton C., Bancroft J.D. (2019). Immunohistochemical techniques. Bancroft’s Theory and Practice of Histological Techniques.

[21] Bima A.I., Mahdi A.S., Al Fayez F.F., Khawaja T.M., Abo El-Khair S.M., Elsamanoudy A.Z. (2021). Cellular Senescence and Vitamin D Deficiency Play a Role in the Pathogenesis of Obesity-Associated Subclinical Atherosclerosis: Study of the Potential Protective Role of Vitamin D Supplementation. Cells.

[22] Aguirre G.A., De Ita J.R., de la Garza R.G., Castilla-Cortazar I. (2016). Insulin-like growth factor-1 deficiency and metabolic syndrome. J. Transl. Med..

[23] de la Monte S.M., Tong M., Schiano I., Didsbury J. (2017). Improved Brain Insulin/IGF Signaling and Reduced Neuroinflammation with T3D-959 in an Experimental Model of Sporadic Alzheimer’s Disease. J. Alzheimers Dis..

[24] Chesik D., De Keyser J., Wilczak N. (2008). Insulin-like Growth Factor System Regulates Oligodendroglial Cell Behavior: Therapeutic Potential in CNS. J. Mol. Neurosci..

[25] de la Monte S.M., Tong M., Bowling N., Moskal P. (2011). si-RNA inhibition of brain insulin or insulin-like growth factor receptors causes developmental cerebellar abnormalities: Relevance to fetal alcohol spectrum disorder. Mol. Brain.

[26] de la Monte S.M., Wands J.R. (2010). Role of central nervous system insulin resistance in fetal alcohol spectrum disorders. J. Popul. Ther. Clin. Pharmacol. = J. Ther. Popul. Pharmacol. Clin..

[27] Garg N., Thakur S., McMahan C.A., Adamo M.L. (2011). High fat diet induced insulin resistance and glucose intolerance are gender-specific in IGF-1R heterozygous mice. Biochem. Biophys. Res. Commun..

[28] Bassil F., Fernagut P.-O., Bezard E., Meissner W.G. (2014). Insulin, IGF-1 and GLP-1 signaling in neurodegenerative disorders: Targets for disease modification?. Progress Neurobiol..

[29] Bassil F., Canron M.H., Vital A., Bezard E., Li Y., Greig N.H., Gulyani S., Kapogiannis D., Fernagut P.O., Meissner W.G. (2017). Insulin resistance and exendin-4 treatment for multiple system atrophy. Brain.

[30] Petersen M.C., Shulman G.I. (2018). Mechanisms of Insulin Action and Insulin Resistance. Physiol. Rev..

[31] Allard J.B., Duan C. (2018). IGF-Binding Proteins: Why Do They Exist and Why Are There So Many?. Front. Endocrinol..

[32] Boughanem H., Yubero-Serrano E.M., López-Miranda J., Tinahones F.J., Macias-Gonzalez M. (2021). Potential Role of Insulin Growth-Factor-Binding Protein 2 as Therapeutic Target for Obesity-Related Insulin Resistance. Int. J. Mol. Sci..

[33] Xiao Z., Chu Y., Qin W. (2020). IGFBP5 modulates lipid metabolism and insulin sensitivity through activating AMPK pathway in non-alcoholic fatty liver disease. Life Sci..

[34] Kobayashi T., Kaneda A., Kamata K. (2003). Possible involvement of IGF-1 receptor and IGF-binding protein in insulin-induced enhancement of noradrenaline response in diabetic rat aorta. Br. J. Pharmacol..

[35] Heald A., Kaushal K., Siddals K., Rudenski A., Anderson S., Gibson J. (2006). Insulin-like Growth Factor Binding Protein-2 (IGFBP-2) is a Marker for the Metabolic Syndrome. Exp. Clin. Endocrinol. Diabetes.

[36] Olszanecka A., Dragan A., Kawecka-Jaszcz K., Fedak D., Czarnecka D. (2017). Relationships of insulin-like growth factor-1, its binding proteins, and cardiometabolic risk in hypertensive perimenopausal women. Metabolism.

[37] Rojas-Rodriguez R., Lifshitz L.M., Bellve K.D., Min S.Y., Pires J., Leung K., Boeras C., Sert A., Draper J.T., Corvera S. (2015). Human adipose tissue expansion in pregnancy is impaired in gestational diabetes mellitus. Diabetologia.

[38] Minchenko D.O., Tsymbal D.O., Davydov V.V., Minchenko O.H. (2019). Expression of genes encoding IGF1, IGF2, and IGFBPs in blood of obese adolescents with insulin resistance. Endocr. Regul..

[39] Abdulsada M.M., Wilhelm Z.R., Opekun A.R., Devaraj S., Jalal P.K., Mindikoglu A.L. (2020). The effect of four-week intermittent fasting from dawn to sunset on circulating brain-derived neurotrophic factor levels in subjects with metabolic syndrome and healthy subjects. Metabol. Open.

[40] Lee I.T., Wang J.-S., Fu C.-P., Lin S.-Y., Sheu W.H.-H. (2016). Relationship between body weight and the increment in serum brain-derived neurotrophic factor after oral glucose challenge in men with obesity and metabolic syndrome: A prospective study. Medicine.

[41] Motamedi S., Karimi I., Jafari F. (2017). The interrelationship of metabolic syndrome and neurodegenerative diseases with focus on brain-derived neurotrophic factor (BDNF): Kill two birds with one stone. Metab. Brain Dis..

[42] Jamali A., Shahrbanian S., Morteza Tayebi S. (2020). The Effects of Exercise Training on the Brain-Derived Neurotrophic Factor (BDNF) in the Patients with Type 2 Diabetes: A Systematic Review of the Randomized Controlled Trials. J. Diabetes Metab. Disord..

[43] Stålnacke B.-M., Sojka P. (2008). Repeatedly Heading a Soccer Ball Does Not Increase Serum Levels of S-100B, a Biochemical Marker of Brain Tissue Damage: An Experimental Study. Biomark. Insights.

[44] Hamed S.A., Hamed E.A., Zakary M.M. (2009). Oxidative stress and S-100B protein in children with bacterial meningitis. BMC Neurol..

[45] Bjursten S., Pandita A., Zhao Z., Fröjd C., Ny L., Jensen C., Ullerstam T., Jespersen H., Borén J., Levin M. (2021). Early rise in brain damage markers and high ICOS expression in CD4+ and CD8+ T cells during checkpoint inhibitor-induced encephalomyelitis. J. Immunother. Cancer.

[46] Huang Y., Li X., Zhang X., Tang J. (2020). Oxymatrine Ameliorates Memory Impairment in Diabetic Rats by Regulating Oxidative Stress and Apoptosis: Involvement of NOX2/NOX4. Oxid. Med. Cell Longev..

[47] Maciejczyk M., Żebrowska E., Nesterowicz M., Żendzian-Piotrowska M., Zalewska A. (2022). α-Lipoic Acid Strengthens the Antioxidant Barrier and Reduces Oxidative, Nitrosative, and Glycative Damage, as well as Inhibits Inflammation and Apoptosis in the Hypothalamus but Not in the Cerebral Cortex of Insulin-Resistant Rats. Oxid. Med. Cell Longev..

[48] Nuzzo D., Galizzi G., Amato A., Terzo S., Picone P., Cristaldi L., Mulè F., Di Carlo M. (2020). Regular Intake of Pistachio Mitigates the Deleterious Effects of a High Fat-Diet in the Brain of Obese Mice. Antioxidants.

[49] Rahmati M., Keshvari M., Mirnasouri R., Chehelcheraghi F. (2021). Exercise and Urtica dioica extract ameliorate hippocampal insulin signaling, oxidative stress, neuroinflammation, and cognitive function in STZ-induced diabetic rats. Biomed. Pharmacother..

[50] Feng C., Jiang Y., Li S., Ge Y., Shi Y., Tang X., Le G. (2022). Methionine Restriction Improves Cognitive Ability by Alleviating Hippocampal Neuronal Apoptosis through H19 in Middle-Aged Insulin-Resistant Mice. Nutrients.

[51] Zheng H., Wang R., Qu J. (2016). Effect of different glucose supply conditions on neuronal energy metabolism. Cogn. Neurodyn.

[52] Mu R., Wu X., Yuan D., Zhao J., Tang S., Hong H., Long Y. (2022). Activation of TGR5 Ameliorates Streptozotocin-Induced Cognitive Impairment by Modulating Apoptosis, Neurogenesis, and Neuronal Firing. Oxid. Med. Cell Longev..

[53] Kim N., Chen D., Zhou X.Z., Lee T.H. (2019). Death-Associated Protein Kinase 1 Phosphorylation in Neuronal Cell Death and Neurodegenerative Disease. Int. J. Mol. Sci..

[54] Dienel G.A. (2019). Brain Glucose Metabolism: Integration of Energetics with Function. Physiol. Rev..

[55] Brundel M., Kappelle L.J., Biessels G.J. (2014). Brain imaging in type 2 diabetes. Eur. Neuropsychopharmacol..

[56] Del Bene A., Ciolli L., Borgheresi L., Poggesi A., Inzitari D., Pantoni L. (2015). Is type 2 diabetes related to leukoaraiosis? an updated review. Acta Neurol. Scand..

[57] Willette A.A., Bendlin B.B., Starks E.J., Birdsill A.C., Johnson S.C., Christian B.T., Okonkwo O.C., La Rue A., Hermann B.P., Koscik R.L. (2015). Association of Insulin Resistance With Cerebral Glucose Uptake in Late Middle-Aged Adults at Risk for Alzheimer Disease. JAMA Neurol..

[58] Berhane F., Fite A., Daboul N., Al-Janabi W., Msallaty Z., Caruso M., Lewis M.K., Yi Z., Diamond M.P., Abou-Samra A.-B. (2015). Plasma Lactate Levels Increase during Hyperinsulinemic Euglycemic Clamp and Oral Glucose Tolerance Test. J. Diabetes Res..

[59] Pelvig D.P., Pakkenberg H., Stark A.K., Pakkenberg B. (2008). Neocortical glial cell numbers in human brains. Neurobiol. Aging.

[60] Ding X., Jian T., Wu Y., Zuo Y., Li J., Lv H., Ma L., Ren B., Zhao L., Li W. (2019). Ellagic acid ameliorates oxidative stress and insulin resistance in high glucose-treated HepG2 cells via miR-223/keap1-Nrf2 pathway. Biomed. Pharmacother..

[61] de la Monte S.M. (2017). Insulin Resistance and Neurodegeneration: Progress Towards the Development of New Therapeutics for Alzheimer’s Disease. Drugs.

[62] Roberts R.O., Knopman D.S., Cha R.H., Mielke M.M., Pankratz V.S., Boeve B.F., Kantarci K., Geda Y.E., Jack C.R., Petersen R.C. (2014). Diabetes and elevated hemoglobin A1c levels are associated with brain hypometabolism but not amyloid accumulation. J. Nucl. Med..

[63] Ashtary-Larky D., Bagheri R., Bavi H., Baker J.S., Moro T., Mancin L., Paoli A. (2022). Ketogenic diets, physical activity and body composition: A review. Br. J. Nutr..

[64] Nuwaylati D., Eldakhakhny B., Bima A., Sakr H., Elsamanoudy A. (2022). Low-Carbohydrate High-Fat Diet: A SWOC Analysis. Metabolites.

[65] Montella L., Sarno F., Altucci L., Cioffi V., Sigona L., Di Colandrea S., De Simone S., Marinelli A., Facchini B.A., De Vita F. (2021). A Root in Synapsis and the Other One in the Gut Microbiome-Brain Axis: Are the Two Poles of Ketogenic Diet Enough to Challenge Glioblastoma?. Front. Nutr..

[66] Lilamand M., Mouton-Liger F., Di Valentin E., Sànchez Ortiz M., Paquet C. (2022). Efficacy and Safety of Ketone Supplementation or Ketogenic Diets for Alzheimer’s Disease: A Mini Review. Front. Nutr..

[67] Walsh J.J., Myette-Côté É., Little J.P. (2020). The Effect of Exogenous Ketone Monoester Ingestion on Plasma BDNF During an Oral Glucose Tolerance Test. Front. Physiol..

[68] Walsh J.J., Neudorf H., Little J.P. (2021). 14-Day Ketone Supplementation Lowers Glucose and Improves Vascular Function in Obesity: A Randomized Crossover Trial. J. Clin. Endocrinol. Metab..

[69] Kackley M.L., Buga A., Crabtree C.D., Sapper T.N., McElroy C.A., Focht B.C., Kraemer W.J., Volek J.S. (2022). Influence of Nutritional Ketosis Achieved through Various Methods on Plasma Concentrations of Brain Derived Neurotropic Factor. Brain Sci..

[70] Lan Y., Huang Z., Jiang Y., Zhou X., Zhang J., Zhang D., Wang B., Hou G. (2018). Strength exercise weakens aerobic exercise-induced cognitive improvements in rats. PLoS ONE.

[71] Park C.H., Kwak Y.-S. (2017). Analysis of energy restriction and physical activity on brain function: The role of ketone body and brain-derived neurotrophic factor. J. Exerc. Rehabil..

[72] Vizuete A.F., de Souza D.F., Guerra M.C., Batassini C., Dutra M.F., Bernardi C., Costa A.P., Goncalves C.A. (2013). Brain changes in BDNF and S100B induced by ketogenic diets in Wistar rats. Life Sci..

[73] Murugan M., Boison D. (2020). Ketogenic diet, neuroprotection, and antiepileptogenesis. Epilepsy Res..

[74] Ziegler D.R., Oliveira D.L., Pires C., Ribeiro L., Leite M., Mendez A., Goncalves D., Tramontina F., Portela L.V., Wofchuk S.T. (2004). Ketogenic diet fed rats have low levels of S100B in cerebrospinal fluid. Neurosci. Res..

[75] Jiang Z., Yin X., Wang M., Chen T., Wang Y., Gao Z., Wang Z. (2022). Effects of Ketogenic Diet on Neuroinflammation in Neurodegenerative Diseases. Aging Dis..

[76] Harun-Or-Rashid M., Inman D.M. (2018). Reduced AMPK activation and increased HCAR activation drive anti-inflammatory response and neuroprotection in glaucoma. J. Neuroinflamm..

[77] Guo M., Wang X., Zhao Y., Yang Q., Ding H., Dong Q., Chen X., Cui M. (2018). Ketogenic Diet Improves Brain Ischemic Tolerance and Inhibits NLRP3 Inflammasome Activation by Preventing Drp1-Mediated Mitochondrial Fission and Endoplasmic Reticulum Stress. Front. Mol. Neurosci..

[78] Wang H., Qi W., Zou C., Xie Z., Zhang M., Naito M.G., Mifflin L., Liu Z., Najafov A., Pan H. (2021). NEK1-mediated retromer trafficking promotes blood-brain barrier integrity by regulating glucose metabolism and RIPK1 activation. Nat. Commun..

[79] Zhu Y., Tang X., Cheng Z., Dong Q., Ruan G. (2022). The Anti-Inflammatory Effect of Preventive Intervention with Ketogenic Diet Mediated by the Histone Acetylation of mGluR5 Promotor Region in Rat Parkinson’s Disease Model: A Dual-Tracer PET Study. Park. Dis..

[80] Sourbron J., Klinkenberg S., van Kuijk S.M.J., Lagae L., Lambrechts D., Braakman H.M.H., Majoie M. (2020). Ketogenic diet for the treatment of pediatric epilepsy: Review and meta-analysis. Child’s Nerv. Syst..

[81] Tao Y., Leng S.X., Zhang H. (2022). Ketogenic Diet: An Effective Treatment Approach for Neurodegenerative Diseases. Curr. Neuropharmacol..

[82] Dyńka D., Kowalcze K., Paziewska A. (2022). The Role of Ketogenic Diet in the Treatment of Neurological Diseases. Nutrients.

[83] Davis J.J., Fournakis N., Ellison J. (2020). Ketogenic Diet for the Treatment and Prevention of Dementia: A Review. J. Geriatr. Psychiatry Neurol..

[84] Mayengbam S., Ellegood J., Kesler M., Reimer R.A., Shearer J., Murari K., Rho J.M., Lerch J.P., Cheng N. (2021). A ketogenic diet affects brain volume and metabolome in juvenile mice. NeuroImage.

[85] Altayyar M., Nasser J.A., Thomopoulos D., Bruneau M. (2022). The Implication of Physiological Ketosis on The Cognitive Brain: A Narrative Review. Nutrients.

[86] Joseph A., Parvathy S., Varma K.K. (2021). Hyperinsulinemia Induced Altered Insulin Signaling Pathway in Muscle of High Fat- and Carbohydrate-Fed Rats: Effect of Exercise. J. Diabetes Res..

[87] Yang L., Li F., Ge W., Mi C., Wang R., Sun R. (2010). Protective effects of naloxone in two-hit seizure model. Epilepsia.

[88] Ghoneim F.M., Khalaf H.A., Elsamanoudy A.Z., Abo El-Khair S.M., Helaly A.M., Mahmoud E.H.M., Elshafey S.H. (2015). Protective effect of chronic caffeine intake on gene expression of brain derived neurotrophic factor signaling and the immunoreactivity of glial fibrillary acidic protein and Ki-67 in Alzheimer’s disease. Int. J. Clin. Exp. Pathol..

[89] Abo El-Khair S.M., Ghoneim F.M., Shabaan D.A., Elsamanoudy A.Z. (2020). Molecular and ultrastructure study of endoplasmic reticulum stress in hepatic steatosis: Role of hepatocyte nuclear factor 4alpha and inflammatory mediators. Histochem. Cell Biol..

[90] Juárez-Vázquez C.I., Gurrola-Díaz C.M., Vargas-Guerrero B., Domínguez-Rosales J.A., Rodriguez-Ortiz J.F., Barros-Núñez P., Flores-Martínez S.E., Sánchez-Corona J., Rosales-Reynoso M.A. (2018). Insulin glargine affects the expression of Igf-1r, Insr, and Igf-1 genes in colon and liver of diabetic rats. Iran. J. Basic. Med. Sci..

[91] Yang Y., Jiang W., Yang S., Qi F., Zhao R. (2020). Transgenerational Inheritance of Betaine-Induced Epigenetic Alterations in Estrogen-Responsive IGF-2/IGFBP2 Genes in Rat Hippocampus. Mol. Nutr. Food Res..

[92] He X., Sun J., Huang X. (2018). Expression of caspase-3, Bax and Bcl-2 in hippocampus of rats with diabetes and subarachnoid hemorrhage. Exp. Ther. Med..

[93] Ghoneim F.M., Abo-Elkhair S.M., Elsamanoudy A.Z., Shabaan D.A. (2021). Evaluation of Endothelial Dysfunction and Autophagy in Fibromyalgia-Related Vascular and Cerebral Cortical Changes and the Ameliorative Effect of Fisetin. Cells.

